# Minimally invasive intrasulcular tunneling technique for treatment of gingival recessions: A case series

**DOI:** 10.1002/ccr3.5699

**Published:** 2022-04-05

**Authors:** Dler Ali Khursheed, Faraedon M. Zardawi

**Affiliations:** ^1^ 275719 Department of Periodontics College of Dentistry University of Sulaimani Sulaymaniyah Iraq

**Keywords:** advanced flap, connective tissue graft, modification of tunneling, tunneling procedure

## Abstract

Coronally advanced flap (CAF) by tunneling procedure was applied on four cases of gingival recession. Post‐operative follow‐up, at different time breaks, recorded full coverage of almost all receded root surfaces. The technique and the clinical outcome of this technique will be demonstrated in this case series report.

## Graphical abstract

Coronally advanced flap (CAF) by tunneling procedure is considered one of the effective root coverage procedures, particularly when it is combined with CTG, in modification of the conventional technique. Post‐operative follow up, at different time breaks, recorded full coverage of almost all receded root surfaces.

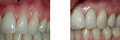

## 1 BACKGROUND

Localized or generalized location of gingival margin apical to cementoenamel junction (CEJ) under different circumstances and etiologic backgrounds called gingival recession.^1^, ^2^ These cause decisive problems such as esthetic, phonetic, impeding plaque control by pulling the gingival margin, and hypersensitivity.^3^ These problems often call for either surgical or nonsurgical treatment. The ultimate goal of surgical procedures is to achieve complete and predictable root coverage.^2^ Several surgical approaches have been accredited for root coverage,^4^ including free gingival graft (FGG) and, connective tissue grafts (CTG) in combination with different flap designs.^5^, ^6^, ^7^ Coronally advanced flap (CAF) combined with CTG is considered an effective root coverage procedure.^7^ Further, CAF could also be performed without vertical incisions and gingival papillary involvement, through tunneling procedure (TUN).^8^, ^9^ Surgical tunneling procedure may be accomplished with combined partial and full thickness flap; however, full thickness flap may be appropriate, especially for thin gingival biotypes.^6^, ^8^ Flap tension should also be minimized to enhance both flap and CTGstability over the denuded roots.^10^ To achieve and maintain appropriate placement of the flap and grafting together during the healing phase, multiple suturing techniques could be adopted for TUN, including sling, suspended, and anchored sutures, to enable the flap and the graft to be sutured and coronally positioned together, or they could be managed separately. Flap undermining and muscle dissection are essential for immobilization of the graft.^11^ Therefore, this case series aimed to present the predictability of covering recession with intrasulcular tunneling preparation by extension of the flap further laterally and apically using a single tunneling instrument and modification of CTG placement by laterally stretched suturing technique.

## 2 CLINICAL PRESENTATION

In addition to demographic data, the following clinical measurements were taken to determine recession types (RT).^12^ Width and length of recessions, clinical attachment level (CAL), vestibular depth from apical extent of the recessions, gingival biotype reference and, further, presence or absence of CEJ ± cervical lesions and tooth mobility were recorded for each individual recession (Table 1).

**TABLE 1:** Clinical evaluation of gingival recession and different postoperative root coverage re‐evaluation assessment

Cases	Age	Sex	Tooth number	Recession length in mm	Recession width in mm	RT	Keratinized tissue width	Gingival thickness	Vestibular depth	CEJ/step	Mobility	Follow‐up (Month)	Root coverage
Case 1	21	Male	21	4	2	2	0	Thin	6	A‐	0	13	CRC
Case 2	47	Female	42	3	3	2	2	Thick	8	A+	0	2	CRC
Case 3	33	Female	42	3	2	1	2	Thin	8	A‐	0	3	CRC
			43	4	3	1	1	Thin	8	A‐	0		CRC
			22	3	2	1	2	Thick	4	A‐	0	2	CRC
			21	3	2	2	1	Thin	3	A‐	0		CRC
			31	3	2	2	2	Thin	2	A‐	0		CRC
			32	2	2	1	2	Thick	3	A‐	0		CRC

All patients were systemically and periodontally fit for the surgery, surgical procedures were performed by one well skilled periodontist. Details of the surgery and the possible complications were explained to the patients, and signed informed consent was taken from them before starting the surgical procedures. Further, the study proposal was submitted to the scientific committee of the college of dentistry, University of Sulaimani, for registration and obtaining ethical approval for undertaking this study.

## 3 CASE MANAGEMENT

**FIGURE 1:**
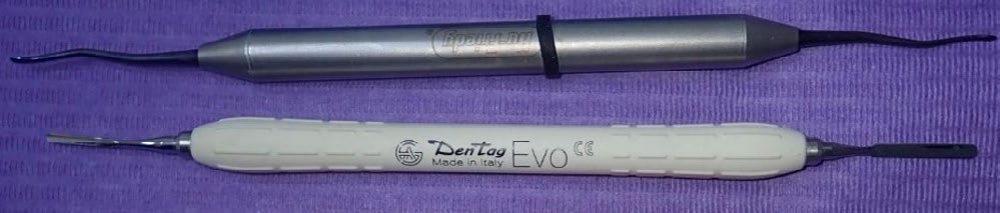
Surgical instrument applied for tunneling procedure

**FIGURE 2:**
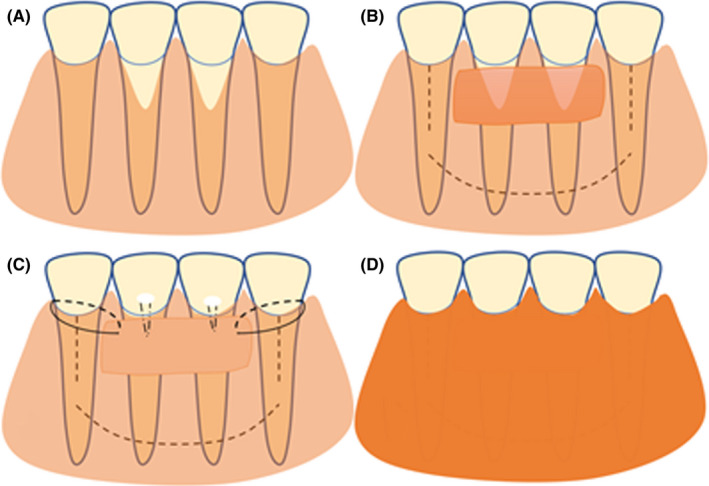
Schematic illustration of modified tunneling procedure: preoperative state (A), dotted lines indicate extensions of the flap, placement of CTG (B), graft is sutured around adjacent teeth, the flap is further advanced coronally, sutured and fixed with composite filling (C), root coverage postoperatively (D)

Root surface debridement was performed with a curette after giving local anesthetics to the area by infiltration. Tunneling was performed using a Hu‐friedy tunneling knife (TKN1 #1 HDL #6) and a fine tissue separator (Dentag Evo) (Figure 1). The procedure was performed by separating the gingival flap and vestibular mucosal tissues from the underlying bone and muscular tissues, respectively. Separation and the entire tunneling preparation were carried out with a single tunneling instrument without using a microblade for sulcular incision. Tunneling started from margins of the recession, extended laterally to mid‐facial line of the adjacent teeth at level of facial CEJ, and extended further apically to minimize tension from underlying muscles (Figure 2B). According to the extent of the recession, FGG of about 1 mm thickness was taken from the molar region of the palate. The donor area was managed by suturing over the hemostatic sponge and the graft was de‐epithelized after harvesting.

The graft was placed under the tunnel and sutured from either side. In the current study, the graft was retained by either 2 circular stitches around adjacent teeth or with composite filling. This was done to hold part of the graft fixed over the receded roots and the other part over the underlying bone, without being sutured to the flap, to retain the graft fixed in place and avoid dislodgment under possible tension. In multiple recession cases, the flap margin was sutured independently and fixed by composite filling to the facial surfaces of teeth (Figure 2C).

## 4 CLINICAL OUTCOMES

**FIGURE 3:**
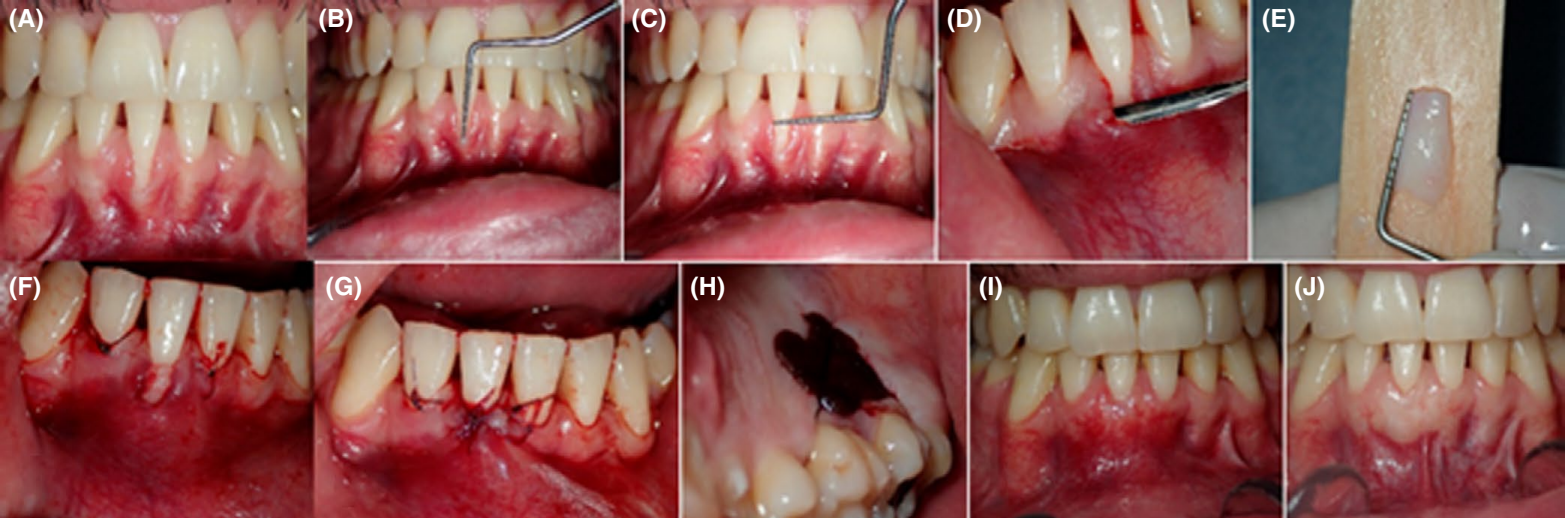
Pre‐operative view RT2 (A), measurement of recession length and width (B and C), extension of the tunnel laterally (D), de‐epithelized free connective tissue graft (E), placement of the graft and suturing laterally (F), advancing the recession margins to cover the exposed graft (G), the donor graft area is sutured with hemostatic sponge (H), CRC 2 weeks and 13 months postoperatively (I and J)

**FIGURE 4:**
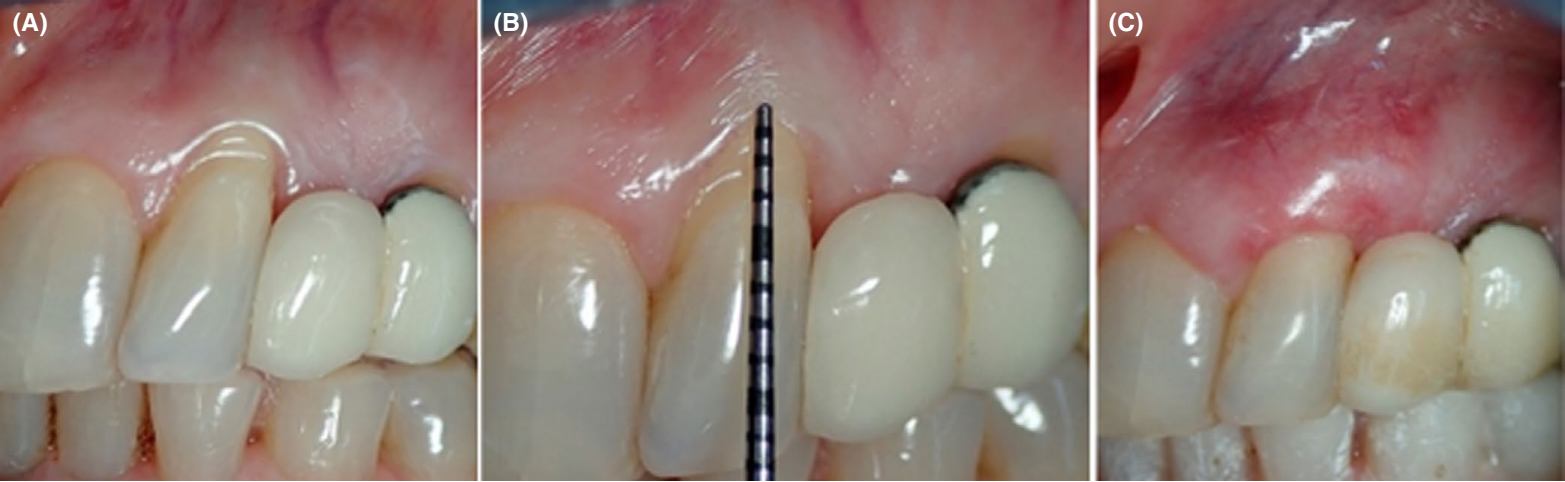
Pre‐operative view RT1 (A), 3 mm gingival recession (B), complete root coverage after 2 months (C)

**FIGURE 5:**
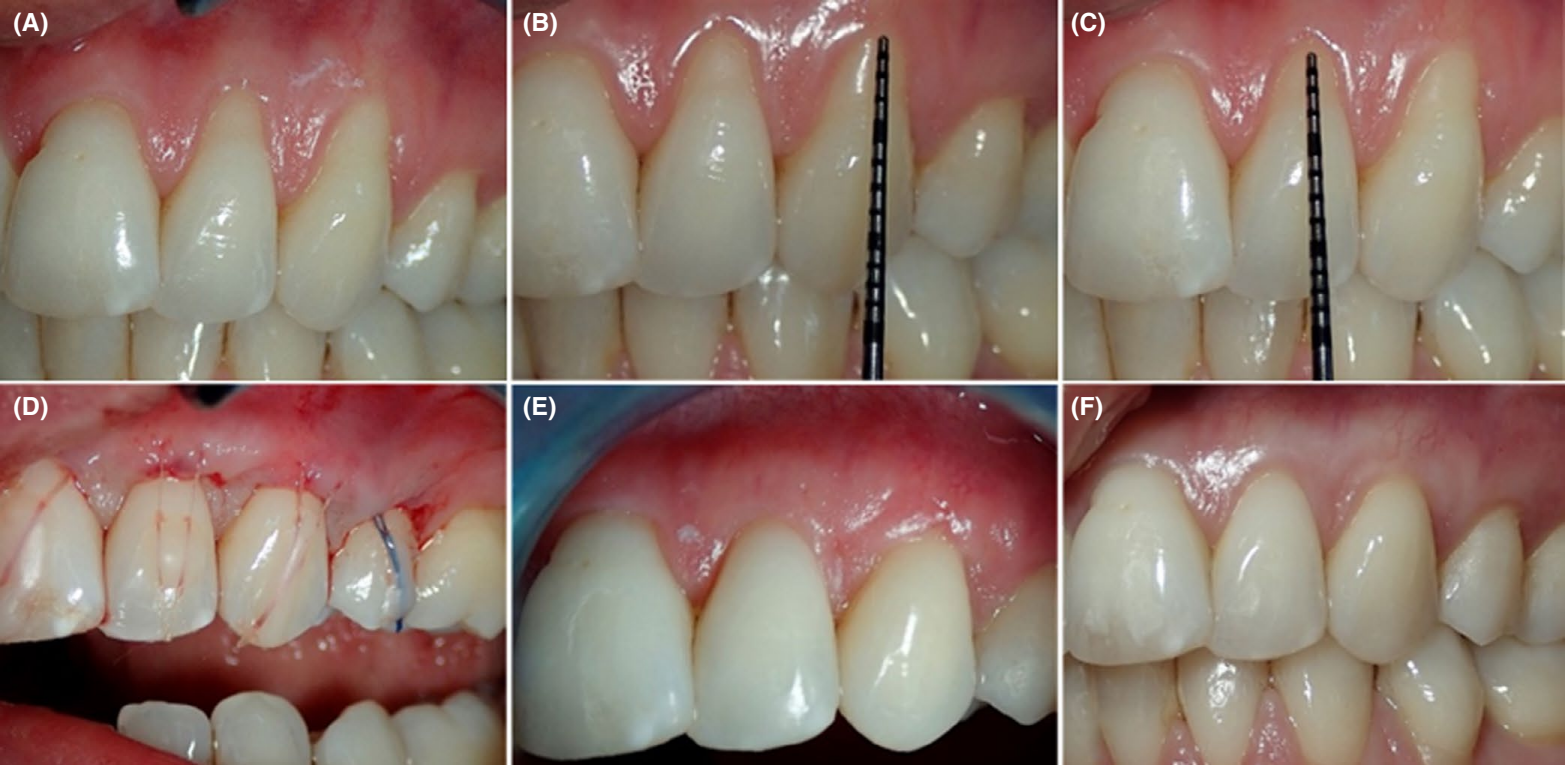
RT1 on teeth # 42 and 43 (A), each showing 3 mm recession (B and C), suturing the flap over the graft (D), complete root coverage after 2 weeks (E), healthy and stable gingiva after 3 months (F)

**FIGURE 6:**
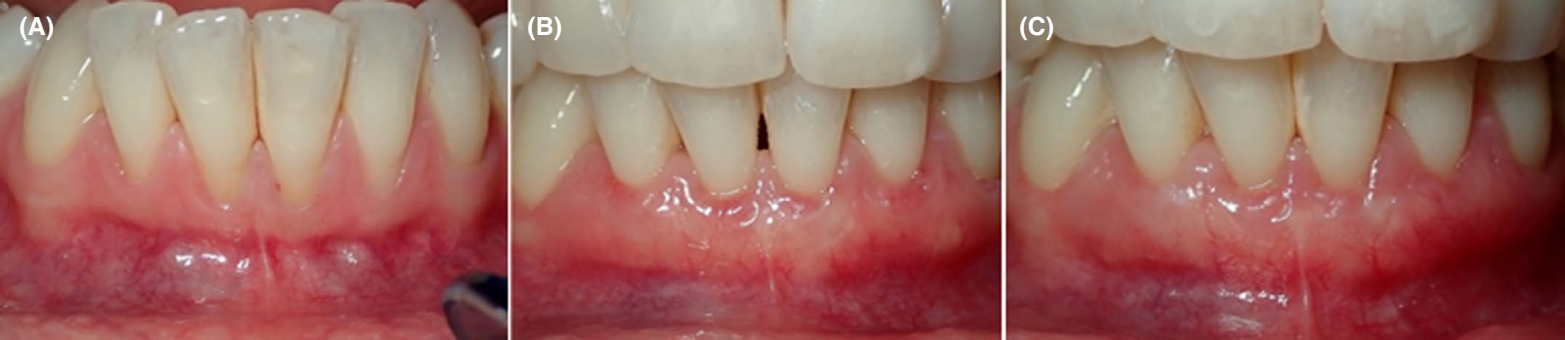
RT2 on teeth #21,31 and RT1 on teeth #22,32 (A), complete root coverage after 2 weeks (B), stable tissue after 2 months (C)

The study included 3 cases, 2 of which were single gingival recession (RT2), Figures 3A–J and 4A–C, and the third case was two multiple recessions on the upper, Figure 5A–F, and lower teeth, Figure 6A–C; all recessions were recorded as RT1 except for #21 and 31 which were recorded as RT2 (Table 1). The recessions were measured at different follow‐up times postoperatively. Complete root coverage was achieved on a total of 8 recessions involved in this study (Table 1).

## 5 DISCUSSION

The incision free design and gingival papillary preservation are considered as the main advantages of TUN.^8^ In the current study, sufficient tissue was undermined with minimum papillary involvement around the recession area^9^ to release active tension from surrounding tissues and approximate gingival recession margins over the CTG. This procedure would also lead to increased blood supply to that part of the graft on the exposed root as no blood supply is obtained from the underlying exposed root.^12^ Furthermore, the remaining part of the graft under the tunnel gets a dual blood supply from the underlying periosteum and overlying subepithelial connective tissue, respectively. This avoids necrosis of the free graft tissue over the denuded root surface by providing dual nutrition for the graft.

In these cases, the flap was further extended on both sides and the apical side to provide a relaxed bed for the graft. Therefore, this method of tissue preparation and CTG suturing may provide high stability of the graft in terms of mechanical and biological aspects. In this procedure, the CTG is completely secured by stretching it laterally and suturing around the teeth adjacent to the recession on both sides without suturing to the underlying periosteum and/or overlying flap tissue. Stretching the graft avoids shrinkage and improves the survival rate by making the graft more responsive to revascularization.^11^, ^13^, ^14^ The only limitation of applying this technique is when thin gingival biotype is present that makes the tunneling preparation with partial thickness flap difficult (Table 2).

**TABLE 2:** Rational for modifying the original technique and the primary limitations of this technique

Why is this case new information?	Minimum papillary involvement with further lateral and apical extension of the tunnel to reduce tension on the coronally advanced flap and the graft The graft was held in place without being sutured to the flap or underlying periosteum by laterally extended sutures around the adjacent teeth.
What are the keys to successful management of this case?	Tension free coronally advancement flap Graft stabilization
What are the primary limitations to success in this case?	There are some limitations in cases of Thin gingival biotype

Higher predictability rate of the clinical outcome could be expected with the current procedure, while the tunnel flap and the graft are relaxed and not under tension to avoid any mobility of the graft that compromises the normal healing process.

## 6 CONCLUSION

With limited number of cases presented in this manuscript, the tunneling procedure for root coverage showed high predictable positive outcomes.

## CONFLICTS OF INTEREST

No conflict of interest was declared by the authors of this study.

## AUTHOR CONTRIBUTION

DK involved in design and case documentation. FZ involved in manuscript preparation and final approval of the manuscript.

## CONSENT

Written informed consent was obtained from the patient to publish this report in accordance with the journal's patient consent policy.
